# Acute Hemorrhagic Edema of Infancy: A Rare Cause of Bilateral Ear Erythema

**DOI:** 10.7759/cureus.82333

**Published:** 2025-04-15

**Authors:** Naima Rasul, Russell Delaney

**Affiliations:** 1 Medical School, Edward Via College of Osteopathic Medicine, Blacksburg, USA; 2 Pediatrics Department, Lewis Gale Medical Center, Salem, USA

**Keywords:** acute edema, ear pain, henoch-schönlein purpura, leukocytoclastic vasculitis, palpable purpura, perichondritis, rash in children, relapsing polychondritis clinical picture, steroid-induced hiccups, steroid treatment

## Abstract

Acute hemorrhagic edema of infancy (AHEI) is a rare cutaneous leukocytoclastic small-vessel vasculitis presenting with purpuric skin plaques on the face, ears, and extremities, frequently associated with fever and edema. Although the condition usually self-resolves over one to three weeks, it can be mistaken for more serious conditions, leading to unnecessary procedures and treatments. This case involves a nine-month-old infant who developed bilateral ear erythema and edema two days after a viral illness and was hospitalized for clinical suspicion of relapsing polychondritis. Within a couple of hours, the infant’s erythema and edema progressed, with the appearance of small papules scattered across the right half of the face. Over five days, the patient developed purpuric lesions on the cheeks, chin, bilateral extremities, and trunk. During the hospitalization, the infant received empiric antibiotics, steroids, and antihistamines. Based on the acute edema, scattered purpuric lesions in the setting of a recent viral illness, and negative infectious workup, the infant was diagnosed with AHEI and subsequently managed with steroids to reduce the inflammation rapidly and antihistamines to reduce corticosteroid-induced gastric complications. The ear erythema, ear edema, and purpuric lesions showed resolution 10 days after initial presentation at the outpatient pediatric follow-up. However, while the infant was in good health overall at the outpatient dermatology follow-up approximately three weeks after hospitalization, he did have residual macular spots on the cheeks and bilateral lower extremities. The macular spots disappeared, and the infant had no cutaneous lesions two months after the initial presentation. The resolution of symptoms after oral steroids confirmed the clinical diagnosis of AHEI rather than relapsing polychondritis.

This case serves to illustrate an unusual presentation of AHEI to aid in the future diagnosis of infantile rashes and to differentiate this benign diagnosis from other, life-threatening conditions. Early recognition of AHEI could prevent unnecessary treatments or procedures in the future.

## Introduction

Acute hemorrhagic edema of infancy (AHEI) is a rare cutaneous leukocytoclastic small-vessel vasculitis typically affecting children under two years of age [1,2]. There have been 458 individually documented cases published in the literature between 1970 and 2019 [2]. AHEI is a self-limiting condition that presents with sudden, localized purpura on the face, ears, and extremities, often associated with fever and edema [2-4]. Diagnosis is based on clinical manifestations but can be supported by a skin biopsy as necessary. A recent retrospective study of 69 children across 22 hospitals in France validated a 1996 study’s clinical criteria of age less than two years, purpuric or ecchymotic targetoid skin lesions with edema on the face, ears, and extremities, lack of visceral involvement, and spontaneous recovery within a few days or weeks [4,5].

The same retrospective study reported that triggering factors were observed in 70% of patients with probable AHEI, with a mean duration of about seven days between onset of trigger and diagnosis [4]. Bacteria, viruses, antibiotics, and vaccinations have been identified as triggers for AHEI [1,4,6-7]. While the exact mechanism of AHEI is not fully understood, the inflammation and necrosis of small blood vessels in the skin are thought to be immune-mediated, triggered by an antigenic stimulus, leading to deposition of immune complexes in the vessel walls [8]. The immune complex deposition activates the complement system, resulting in neutrophil recruitment and release of inflammatory mediators, causing vessel wall damage and increased vascular permeability [8].

Since AHEI can present with additional, deceivingly alarming symptoms such as epistaxis, gastrointestinal hemorrhage, and renal involvement (proteinuria or hematuria) [2,3], the condition is often misdiagnosed, resulting in unnecessary hospitalizations and procedures [4]. Differential diagnoses for AHEI include IgA vasculitis (Henoch-Schönlein purpura (HSP)) [1,4,6], purpura fulminans [1,4], erythema multiforme [1,3], Kawasaki syndrome [1,3], Sweet syndrome [1,3], meningococcemia [1], and drug eruptions [6]. The main differential diagnosis is IgA vasculitis, which differs from AHEI by older patient population (three to eight years of age), location of purpuric lesions (predominantly on lower extremity, sparing face and ears), duration of purpuric lesions (greater than one month), and greater likelihood of systemic findings such as abdominal pain, renal disease, and arthritis [3]. The most concerning diagnoses to differentiate are relapsing polychondritis, perichondritis, child abuse, meningococcemia, dermatologic manifestations of hematologic disease, and septicemia.

An important clinical manifestation that sets AHEI apart from more concerning differential diagnoses, such as meningococcemia and Kawasaki, is the nontoxic appearance of the infant [4,6]. Aside from a low-grade fever, nonpitting edema, and the dramatic skin presentation, the infant is generally in good health [4,6]. The absence of pruritus differentiates AHEI from urticaria [4]. A skin biopsy of AHEI would demonstrate leukocytoclastic vasculitis, but unlike IgA vasculitis, approximately 75% of AHEI cases do not show IgA deposition by direct immunofluorescence [3]. In a systematic review of 75 cases of AHEI, Pellanda et al. reported vessel wall deposition of complement C3 in 40 cases and IgA deposition in 21 cases [3,8].

Treatment is symptomatic, as the condition usually resolves spontaneously over one to three weeks [6,7]. The use of antihistamines and steroids is controversial [6,9-17]. Antihistamines can be used to provide symptomatic relief of itching and angioedema, if present. The purpose of steroids in AHEI is either potential rapid relief of severe manifestations such as generalized edema and discomfort or to empirically treat differential diagnoses such as HSP, Kawasaki disease, erythema multiforme, and relapsing polychondritis [6,9-17]. Depending on the child’s presentation, antibiotics are sometimes started empirically due to concern for meningococcemia and purpura fulminans and then discontinued after cultures return negative [6]. Inpatient care is only required if the diagnosis is in doubt or if another more serious condition remains in the differential [6].

This case involves a nine-month-old male infant who presented to a pediatrician's office for bilateral external ear edema and erythema after an ER visit and then was admitted to the hospital for further evaluation by specialists. A purpuric rash, not present on initial presentation, began to develop over the course of the hospital admission, indicating an unusual presentation of a rare diagnosis of AHEI.

## Case presentation

A nine-month-old term male infant presented to a pediatrician's office with less than six hours of onset of bilateral ear pain and edema. The infant had developed a nonfebrile barking cough and nasal congestion two days beforehand with a known sick contact that had croup. He was diagnosed with viral croup in the ER and given oral dexamethasone 5 mg the day prior to presentation at the pediatrician’s office. The patient’s upper respiratory symptoms improved with the dexamethasone. The morning after the ER visit, the patient’s mother noticed erythema and mild swelling of the left ear after the patient had been fussy, crying, and unable to sleep. The patient received Tylenol for pain, but the mother denied the development of a fever. At the time of the pediatrician's office evaluation, the patient was afebrile and had edematous and erythematous external ears with mild nasal congestion. The edema extended posteriorly into the mastoid area bilaterally. Physical examination showed increased warmth to the touch and no tenderness to palpation of the ears. The ear canal was non-erythematous. Tympanic membranes were visualized bilaterally, non-erythematous, and non-bulging, with light reflex seen. No rash was noted on the face, trunk, chest, abdomen, or extremities. There were no remarkable abnormalities on the heart, lungs, abdominal, neurological, musculoskeletal, and extremity examinations.

The infant was up to date on required vaccinations for his age. The patient had a history of three episodes of acute otitis media successfully treated with two courses of amoxicillin and one course of amoxicillin-clavulanate. The most recent episode was more than 20 days prior to this presentation.

**Figure 1:**
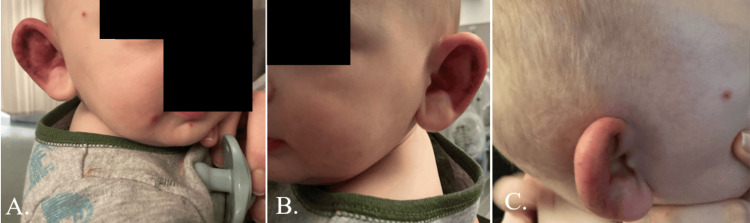
Presentation of the infant to the pediatrician's office on admission day showing three erythematous papules on the right cheek and chin (A) and no lesions on the left (B). Panel C shows the extension of the edema and erythema into the preauricular and temporal regions. These photos were obtained with the permission of the infant’s parent.

The patient was admitted for a three-day hospital stay. Upon presentation to the pediatrician's office an hour later, the patient had developed three erythematous, non-draining, nonpruritic papules on the right cheek and chin (Figure 1A). The edema and erythema had extended to the preauricular and temporal regions of the head (Figure 1C). A consulting otolaryngologist classified these symptoms as potential relapsing polychondritis and initiated an intravenous steroid. A single dose of oral amoxicillin-clavulanate followed by intravenous ampicillin-sulbactam was empirically given for possible infection. An oral nonsteroidal anti-inflammatory drug was given for pain. A consulting pediatric rheumatologist agreed with the otolaryngologist’s concerns and management.

**Figure 2:**
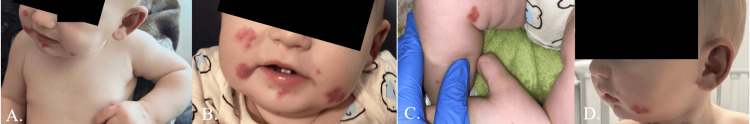
Enlargement and darkening of existing papules and addition of new purpuric plaques on the second (A) and third days (B and C) of hospital admission. Panel C shows the beginning of purpuric plaques appearing on the extremities. Panel D shows improvement in edema and erythema of the ears. These photos were obtained with the permission of the infant’s parent.

On the second day of hospital admission, four new purpuric plaques appeared on the face, while the existing papules had darkened, enlarged in diameter, and flattened (Figure 2A). On the third day, several circular purpuric plaques and papules were noted on bilateral upper and lower extremities with improvement in the edema and erythema of the ears (Figure 2C-2D). There was also a reported decrease in the size of existing purpuric lesions. A consulting dermatologist elected not to biopsy lesions while inpatient since the patient was improving on the current steroid course.

Hospital work-up included posteroanterior and lateral chest radiographs, CBC with differential, comprehensive metabolic panel, peripheral blood smear, C-reactive protein, ESR, urinalysis, antibody panel, complement C3 and C4, and ferritin to evaluate infectious, hematologic, and inflammatory etiologies. A normal WBC count ruled out infection. CRP, ESR, antibody panel, and complements were ordered to evaluate autoimmune etiologies. The patient had low complement C4, weakly positive antinuclear antibody, and positive SCL-70 antibody. In the absence of other clinical signs of scleroderma, the pediatric rheumatologist interpreted these labs as insignificant and likely to indicate an autoinflammatory rather than an autoimmune condition. The CRP level was slightly elevated for the first two days of hospitalization at 1.0 and 2.1, respectively, and normalized on the third day of hospitalization. The ESR level was normal at 11. Normal ESR levels with mild elevation in serum CRP markers reduced clinical suspicion of relapsing polychondritis. Radiographic imaging of the chest was significant for mild perihilar peribronchial thickening, typically seen in lower respiratory tract viral infection or reactive airways disease.

**Figure 3:**
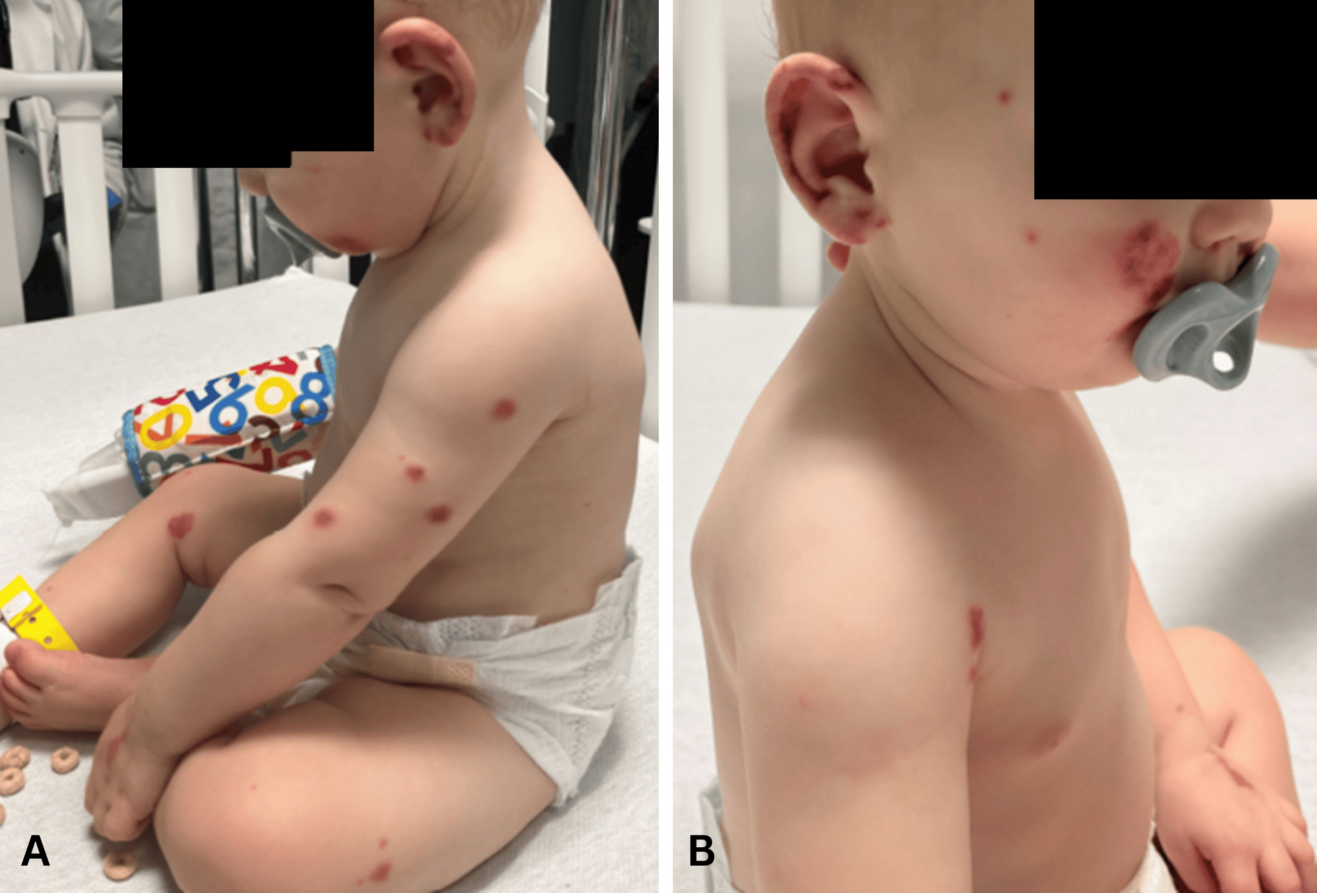
(A and B) Day of hospital discharge showing purpuric plaques on the face and bilateral upper and lower extremities while sparing the trunk. New purpuric lesions also appeared on the infant’s ears. These photos were obtained with the permission of the infant’s parent.

The absence of other diagnostic criteria such as polyarthritis, nasal chondritis, ocular inflammation, or respiratory tract chondritis makes relapsing polychondritis less likely. Based on the acute edema, lack of systemic manifestations, and scattered purpuric lesions in the setting of a recent viral illness with negative infectious and autoimmune workups, the infant was discharged with a clinical diagnosis of AHEI (Figure 3).

During the hospitalization, the patient was managed with steroids to reduce the inflammation and edema rapidly and antihistamines to reduce the risk of corticosteroid-induced gastric complications. The patient was scheduled for follow-up appointments with a pediatrician, dermatologist, and allergist. The infant was discharged with a 12-day oral prednisolone taper to continue reducing the edema and oral famotidine 8 mg twice daily until completion of steroid treatment for gastrointestinal prophylaxis. The prednisolone was dosed at 1.5 mL twice daily for one day, then 1 mL twice daily for four days, 0.75 mL twice daily for four days, and 0.5 mL twice daily for four days.

Clinic follow-up with the allergist five days after initial presentation revealed new purpuric plaques on the trunk and lower extremities with continued improvement of initial lesions. The outpatient allergist agreed with the pediatrician's assessment and noted that it could take up to six weeks for complete resolution of the lesions.

At the pediatrician’s office, 10 days after initial presentation, the patient’s skin lesions showed marked improvement with residual macular spots. There were no purpuric lesions at this time. The ear erythema and edema had resolved, and the infant was overall doing well. However, the patient did have small residual macular spots on the cheeks and bilateral lower extremities one month after the initial onset of symptoms at the outpatient dermatology follow-up. The macular spots disappeared, and the infant had no cutaneous lesions two months after the initial presentation. The resolution of symptoms after oral steroids with no relapse six months after hospitalization confirmed the clinical diagnosis of AHEI rather than relapsing polychondritis.

## Discussion

AHEI is a benign type of cutaneous leukocytoclastic vasculitis affecting children under two years of age [1,2]. AHEI is considered a separate entity from HSP due to better prognosis and lower rates of visceral involvement and IgA skin depositions [6]. AHEI is characterized by a triad of fever, edema, and targetoid-shaped purpura over the face, ears, and extremities that usually develops rapidly over 24-48 hours, with most cases occurring in winter months [1,6]. The gradual onset (with new lesions appearing five days after initial onset), transformation of initial papular lesions into plaques, trunk involvement, pattern in appearance of lesions (first ears, then face, then extremities, then trunk), and absence of fever made this nine-month-old male infant’s case an unusual presentation of AHEI. Some characteristic features seen in this case were the infant’s age, edema, purpuric plaques, primary distribution of lesions on the face, ears, and extremities, duration of purpuric lesions and edema less than three weeks [6], nontoxic appearance, preceding viral illness, and occurrence during a winter month. The infant's prognosis, including the residual macular spots, followed the typical course of AHEI cases in the literature [13].

Since AHEI can mimic other conditions, keeping a broad differential and evaluating for life- or limb-threatening conditions is important. Relapsing polychondritis and perichondritis were the most concerning differentials in this case because of the initial presentation with erythema, edema of the external ear, and history of recurrent ear infections. This infant was admitted to the hospital to be evaluated by an otolaryngologist for these conditions. If untreated, relapsing polychondritis can lead to inflammation of cartilaginous structures, saddle nose and cauliflower ear deformities, and airway collapse [6]. Untreated perichondritis can result in abscess formation, cartilage necrosis, and subsequent deformities [6].

According to an observational study of 18 patients with relapsing polychondritis, the most sensitive diagnostic criteria of the three most widely recognized were the Damiani and Levine criteria [18]. In the Damiani and Levine criteria, a diagnosis of relapsing polychondritis is made when either three or more of the McAdam's criteria are present (bilateral auricular chondritis, nonerosive seronegative inflammatory polyarthritis, nasal chondritis, ocular inflammation (e.g., conjunctivitis, keratitis, scleritis, uveitis), respiratory tract chondritis (e.g., laryngeal, tracheal, or bronchial), audiovestibular damage (e.g., hearing loss, vertigo)), or one or more of the McAdam's criteria are present along with histologic confirmation, or there is chondritis in two or more separate anatomic locations with response to steroids [18]. Perichondritis classically presents with pain, erythema, and swelling of the auricle, sparing the earlobe, with a history of ear trauma (piercings, surgery, or insect bites). It is diagnosed by positive bacterial cultures [19]. The infant in this case did not meet the Damiani and Levine criteria, lowering the index of suspicion for relapsing polychondritis as a differential and making AHEI the more likely diagnosis. Additionally, in relapsing polychondritis, the median duration before the first recurrence was found to be 202 days (approximately 6.7 months) in a retrospective study of 34 patients at the Kyoto University Hospital [20]. In this case, the infant did not relapse over a six-month period, which is consistent with the prognosis of AHEI [6,8,10,13].

Regarding treatment, there is a consensus in the literature review that steroids and antihistamines do not alter the disease course [6,9-14]. However, some case reports noted improvement of severe manifestations within 24 hours of administering systemic corticosteroids [15-17]. Two case reports noted relapse soon after discontinuation of the steroid, followed by improvement upon re-initiation of the steroid [16-17]. A case report of an 11-month-old male who presented with fever, swelling, and papular lesions on the left foot and was initially diagnosed as necrotizing fasciitis was later found to have histopathological findings suggestive of AHEI and treated with supportive care only, with spontaneous resolution of lesions over several weeks [14]. Although recurrence is possible, the prognosis of infants with AHEI is generally excellent, with recovery reported to take anywhere from one week up to 35 days [6].

## Conclusions

AHEI is a rare cutaneous leukocytoclastic small-vessel vasculitis typically affecting children under two years of age. The most common manifestation of AHEI is a triad of fever, edema, and purpuric plaques over the face, ears, and extremities. AHEI is commonly associated with an infectious, environmental, or iatrogenic trigger. The symptoms can be deceivingly alarming, resulting in unnecessary hospitalizations and procedures. Treatment is symptomatic, as the condition usually resolves spontaneously over a few weeks.

The gradual onset (with new lesions appearing five days after initial onset), initial papular appearance of lesions, trunk involvement, pattern in development of lesions (first ears, then face, then extremities, then trunk), and absence of fever made this case atypical. This case demonstrates that AHEI can be challenging to differentiate from conditions such as relapsing polychondritis and perichondritis, and therefore, a thorough evaluation is recommended. If the diagnosis is unclear, empiric treatment for life-threatening conditions may be initiated until the diagnosis becomes more transparent. We hope this case can inform future diagnoses of infants with AHEI by illustrating an unusual presentation of this rare diagnosis. While several case reports noted rapid improvements with steroids, the overall evidence is conflicting, with the edema and purpuric lesions in this case taking 10 days to resolve after steroid initiation. Thus, we suggest that future studies explore the benefits and outcomes of steroid management versus supportive care.
